# Sub-Optimal Nutritional Knowledge and Dietary Intake in Gaelic Team Sport Athletes: Limited Associations Between Knowledge and Dietary Adequacy

**DOI:** 10.3390/nu18142259

**Published:** 2026-07-10

**Authors:** Christopher V. McDonald, Emeir M. McSorley, Andrea M. McNeilly, Pamela J. Magee

**Affiliations:** 1 Nutrition Innovation Centre for Food and Health (NICHE), School of Biomedical Sciences, Ulster University, Coleraine BT52 1SA, UK; mcdonald-c28@ulster.ac.uk (C.V.M.); em.mcsorley@ulster.ac.uk (E.M.M.); 2 School of Sport and Exercise Sciences, Ulster University, York St., Belfast BT15 1ED, UK; a.mcneilly@ulster.ac.uk

**Keywords:** sports nutrition, team sports, Gaelic football, hurling, camogie

## Abstract

**Background/Objectives:** To assess the nutritional knowledge and dietary intake of Gaelic team sport athletes and investigate any association between nutrition knowledge and dietary intake. **Methods:** Male/female athletes competing in Gaelic football, hurling and camogie, aged 18–40 years and training/competing at least twice weekly, were recruited. Nutrition knowledge was assessed using the validated Nutrition for Sport Knowledge Questionnaire (NSKQ). Dietary intake was assessed using a 4-day semi-quantitative food diary. **Results:** In total, 354 players (male *n* = 215, female *n* = 139; elite *n* = 133, sub-elite *n* = 221) completed the NSKQ; 53.1% had “poor” knowledge (score ≤49%). Dietary intake was reported by 139 players (39.3%), whose mean knowledge was also “poor” (49.9 ± 13.1%), with scoring patterns identical to non-reporters. Participants scored “average” in weight management, macronutrient, and alcohol subsections, and “poor” in micronutrient, sports nutrition, and supplement subsections. Dietary intake was sub-optimal; 84.9% under-consumed energy (median deficit 755 kcal/d [IQR −1085, −115]), primarily due to insufficient carbohydrate intake (median 3.2 g/kg/d [IQR 2.6, 3.8]; only 20.9% met the 4–8 g/kg/d recommendation). Most participants met protein and fat recommendations, although few met vitamin D or fibre recommendations, and no female participant met the iron recommendation. Those with average or above knowledge were more likely to meet energy and fibre recommendations; no further significant associations were observed. **Conclusions:** These findings suggest that Gaelic team sport athletes lack nutrition knowledge, and their dietary intake appears sub-optimal in several respects. In unadjusted analyses, higher knowledge was modestly associated with meeting energy and fibre recommendations. These findings highlight the need for nutrition interventions, combining education with behaviour change strategies.

## 1. Introduction

Gaelic football, hurling and camogie are amateur team sports played predominantly in Ireland [1], though with a significant presence worldwide, and are governed by the Gaelic Athletic Association (GAA). Most athletes compete at the club level, which is considered sub-elite. Top performing male and female club players can be selected to represent their county, where the demands of both training and competition are considered elite level [2]. These team sports are all classified as invasion games, imposing numerous technical and tactical demands on athletes alongside physical demands such as evasion, high-velocity running, agility, strength, and power [3]. They are multidirectional and intermittent in nature, requiring athletes to perform repeated high-intensity sprints interspersed with prolonged aerobic work [3,4].

Existing evidence indicates that team sport athletes generally demonstrate sub-optimal nutritional knowledge [5]; this has been observed in Gaelic team sport athletes including; Hurlers, Camogie players and female and male [6,7,8,9] Gaelic footballers. Nutrition knowledge appears consistently low irrespective of playing level, with both elite and sub-elite team sport athletes [7,8,9] failing on average to achieve “good” or “excellent” levels of nutrition knowledge. The dietary intake of Gaelic team sport athletes has also been observed to be sub-optimal for the demands of their sport [8,10] and, although the evidence in GAA sports specifically is limited, especially with female athletes, some consistent dietary intake patterns are emerging. Commonly, Gaelic team sport athletes are under-consuming overall energy; this deficit typically arises from sub-optimal intakes of carbohydrates. In the absence of sport-specific carbohydrate guidelines for GAA athletes, recommendations for team sport athletes from comparable field sports (4–8 g/kg/d) provide a useful benchmark, one against which existing evidence indicates that Gaelic players consistently fall short [8,10,11]. Unfortunately, without sport-specific guidelines, it is also more difficult to account for factors such as playing position when estimating fuelling demands, as the varying physical demands of each role are not reflected in generic recommendations. Additionally, Gaelic team sport athletes are failing to meet requirements for certain micronutrients such as; vitamin A, vitamin D, selenium, potassium, zinc, magnesium and calcium [8]. This under-fuelling by Gaelic team sport athletes can negatively affect exercise performance by decreasing power output, time to fatigue, work capacity and recovery, and could potentially result in adverse health effects such as causing hypoglycaemia and higher injury risk [11,12]. Furthermore, long term under-fuelling exposes athletes to risks associated with low energy availability (LEA) such as overreaching, compromised immune function, impaired bone health, endocrine and cognitive disturbances [13]. It has been proposed that poor nutrition knowledge may contribute to sub-optimal dietary intake, with improvements in nutrition knowledge potentially enhancing dietary behaviours [5]. However, there is currently limited research assessing the relationship between nutrition knowledge and dietary intake in Gaelic team sport athletes; the available evidence is insufficient to draw any firm conclusions [6,8]. Although previous studies have investigated nutritional knowledge or dietary intake in Gaelic athletes, these have typically been examined independently, often within a single Gaelic sport or specific athlete population. Consequently, little is known about whether nutritional knowledge is associated with dietary intake across Gaelic team sports or whether access to nutrition support influences nutritional knowledge. Few studies have simultaneously characterised nutritional knowledge, dietary intake, and access to nutrition support within the same cohort using a validated Sports Nutrition Knowledge Questionnaire in both male and female athletes across multiple Gaelic team sports and playing level. Addressing these gaps may provide a more comprehensive understanding of the nutritional challenges Gaelic athletes experience and help inform the development of targeted nutrition education interventions. Therefore, the aim of this study was to assess nutritional knowledge and dietary intake in male and female athletes participating in traditional Gaelic team sports (Gaelic football, hurling, and camogie) using the validated Nutrition for Sport Knowledge Questionnaire (NSKQ) and a four-day food diary. A secondary aim was to examine the associations between nutritional knowledge, dietary intake, participant characteristics, and access to nutrition support. Nutrition knowledge in the context of this study is defined as the player’s level of accurate comprehension of specific sport nutrition topics, quantified by their overall Nutrition for Sport Knowledge Questionnaire (NSKQ) total score and score in each subsection.

It was hypothesised that team sport athletes will have sub-optimal nutrition knowledge as determined by the validated NSKQ and sub-optimal dietary intake in relation to the demands of their sport, as determined by sports nutrition recommendations. Team sport athletes with higher nutritional knowledge scores were hypothesised to be more likely to meet current sports nutrition recommendations.

## 2. Materials and Methods

A cross-sectional, observational design was used, and participants were recruited and asked to complete the NSKQ via the online platform Research Electronic Data Capture (REDCap) software (v16.0.37, Vanderbilt University, Nashville, TN, USA) and a 4-day food diary (either electronically or in hardcopy). This research was conducted in accordance with the Declaration of Helsinki and was approved by Ulster University’s School of Biomedical Sciences Ethics Filter Committee (FCBMS-19-017). Informed consent was obtained from all participants.

Power analysis indicated that a minimum of 95 participants was required; the final sample met this requirement. A substantially larger sample was intentionally recruited to account for anticipated attrition during completion of the 4-day food diary and the expected prevalence of dietary misreporting, thereby ensuring an adequately powered dietary intake sample. The sample size for the study was based on the findings of McCrink et al. (2021) [8], where mean NSKQ score in Gaelic footballers was reported to be 40.2 ± 12.4%. It was calculated that 95 players are needed to achieve a 95% CI width of ±2.5%. As the study aimed to examine the relationship between nutrition knowledge and dietary intake, a larger sample was recruited to account for failure to complete the 4-day food diary in addition to completing the NSKQ and to account for dietary misreporting. As such, 354 players were recruited to the study, with 139 participants completing both the NSKQ and 4-day food diary.

### 2.1. Participants

Participants were recruited through social media and through contacting teams directly. Means of contact included in person conversations, emails and phone calls to team management. Interested athletes contacted the researcher and were then screened for suitability based on study inclusion criteria, i.e., apparently healthy male/female Gaelic team sport athletes competing in Gaelic football, hurling or camogie, aged between 18 and 40 years, training/competing at least twice a week. Participants were recruited from elite (county) and sub-elite playing levels.

### 2.2. Nutrition Knowledge Questionnaire

The validated NSKQ was used to assess nutrition knowledge of the team sport athletes [14,15]. The NSKQ was specifically designed for athlete populations and was developed using a novel eight-step validation framework incorporating both classical test theory and Rasch analysis. The validation process included assessment of face validity, content validity, construct validity, internal reliability, and test–retest reliability, supporting its use as a valid and reliable tool for assessing sports nutrition knowledge in athlete populations [14,15]. The questionnaire comprises 87 questions across six subsections: weight management, macronutrients, micronutrients, sports nutrition, supplementation, and alcohol. One point is awarded for each correct response, with higher scores indicating greater nutrition knowledge.

Demographic questions, adapted to the relevant sport, were included at the beginning of the questionnaire and were used to collect data on age, education level, years playing selected sport and self-reported habitual training volume (hours of training per week). In addition, players were asked about nutrition support provided and the type of nutrition support they would find most useful. The NSKQ takes approximately 25 min to complete and consists of 87 questions, separated into six subsections: weight management (*n* = 12), macronutrients (*n* = 30), micronutrients (*n* = 13), sports nutrition (*n* = 12), supplementation (*n* = 12), and alcohol (*n* = 8). Scoring of the NSKQ was categorised according to Trakman et al. (2018) [16], with scores of 0–49% considered reflective of “poor”, 50–64% “average”, 65–75% “good” and 76–100% “excellent” nutrition knowledge. Due to a low number of participants scoring within the “good” (*n* = 25) or “excellent” (*n* = 7) range, the average, good and excellent nutrition knowledge scores were combined into one category “average and above nutrition knowledge” (50–100%).

### 2.3. Anthropometrics

Height (m) and weight (kg) of the athletes were self-reported within the online questionnaire. Body mass index (BMI) was calculated as: weight (kg)/height (m^2^) [17].

### 2.4. Dietary Intake

Dietary intake was recorded using a 4-day food diary over two weekdays and two weekend days. All participants were given a paper copy or electronic copy of the same blank 4-day food diary and received the same set of comprehensive instructions in relation to completing the food diary, including an example on how to complete a 4-day food diary. Diaries were accepted in multiple formats (food diary hardcopy template, food diary pdf template, hardcopy and online written food diaries, screenshots from diet tracking apps such as MyFitnessPal), provided that participants completed all 4 days of assessment. To quantify reported dietary intake data and enable subsequent analysis, Nutritics (Nutritics Ltd., *Research Edition, v6.00, Dublin, Ireland) nutrition analytic software was used. Median nutrient intake for each day and total average across the 4 days was calculated for energy (kcal/d, KJ/d) and macronutrients; carbohydrate, protein and fat were quantified as absolute intake (g/d), relative to body mass (g/kg/d) and as a proportion of total energy intake (%EI). Dietary intake data were compared to specific United Kingdom (UK) national Daily Recommended Values (DRVs) [18,19,20,21] and sport nutrition recommendations where relevant [11,12].

### 2.5. Misreporting

The literature consensus is that self-reported dietary intake data is typically prone to misreporting, with participants both under- and over-reporting dietary intake. The revised Goldberg cut-off [22] was applied at the group and individual level to identify misreporting in this sample, using the following equations:

$$\begin{array}{c} EI:BMR>PAL\times \exp\left(SD_{min}\times \frac{S/100}{\sqrt{n}}\right)\\ S=\sqrt{\frac{CV_{wEI}^{2}}{d}+CV_{wB}^{2}+CV_{tP}^{2}} \end{array}$$

Cut-offs were derived using ±2 *SD* (95% confidence limits), incorporating within-subject variation in energy intake (*CV_wEI_* = 23%), error associated with estimated BMR (*CV^*2*^_wB_* = 9.8%), between-subject variability in physical activity level (*CV^*2*^_tP_* = 15%), and four days of dietary assessment. Basal metabolic rate was estimated using the Harris–Benedict equation, and a physical activity level (PAL) of 1.6 was assumed. At the group level, an EI:BMR ratio < 1.57 and >1.63 classified the sample as under- and over-reporters, respectively. At the individual level, EI:BMR ratios < 1.25 and >2.06 were used to classify participants as under- and over-reporters. Given the primary aim was to examine the associations between nutrition knowledge and dietary intake, primary analyses were conducted using the full dietary sample; then, to evaluate the impact of misreporting on outcomes, sensitivity analyses were conducted by repeating all dietary intake analyses using the plausible reporters only, identified using the revised Goldberg cut-off method.

### 2.6. Statistical Analysis

Statistical analyses were performed using SPSS (IBM SPSS Statistics for Windows, Version 26.0, IBM Corp., Armonk, NY, USA). Data distribution was evaluated using histograms and Q-Q plots. Normally distributed variables were presented as mean ± SD and analysed using parametric tests. Non-normally distributed variables were presented as median [IQR] and analysed using non-parametric tests. One-way ANOVA was used to compare nutrition knowledge scores between sports. Independent samples *t*-tests were used to compare nutrition knowledge scores between sex and playing level. Chi-squared analysis, or Fisher’s Exact Test where expected cell frequencies were below five, were used to compare the percentage of players meeting dietary recommendations between nutrition knowledge categories (poor nutrition knowledge vs. average and above nutrition knowledge). One-sample Wilcoxon signed-rank tests were used to compare dietary intakes with single-value DRVs and sports nutrition recommendations; for range-based recommendations, no one-sample tests were conducted and the percentage of participants meeting the range was reported descriptively. The level of significance was set at *p* < 0.05.

## 3. Results

### 3.1. Demographics

A total of 354 participants were recruited across 85 teams, (male *n* = 215 (60.7%), female *n* = 139 (39.3%)) with representation from Gaelic football *n* = 271 (76.6%), hurling *n* = 28 (7.9%) and camogie *n* = 55 (15.5%). Participants were recruited across different playing levels, *n* = 133 (37.6%) competed at elite level (county) and *n* = 221 (62.4%) the sub-elite level (club and university). All participants successfully completed the NSKQ and the demographic data of the full cohort is presented in Table 1. Out of the 354 participants who completed the NSKQ, only 139 (39.27%) successfully submitted their 4-day food diary (excluding six additional food diaries which were submitted but were incomplete). Dietary reporters (*n* = 139) and non-reporters (*n* = 215) were compared on age, sex, playing level, weekly training hours, years playing their sport, education level and access to nutritional support. No significant differences were observed for any variable except sex, with female participants significantly underrepresented amongst dietary reporters (25.9% vs. 47.9% of non-reporters (*p* < 0.001)). Of these 139 participants, 78 were identified as potential misreporters at the individual level after the revised Goldberg cut-off method [22] was applied (*n* = 74 under-reporters; *n* = 4 over-reporters). At the group level, the mean EI:BMR ratio of 1.29 fell below the cut-off of 1.57, indicating that the sample as a whole was classified as under-reporters. Primary analyses were conducted using the full dietary sample; to evaluate the impact of misreporting on outcomes, sensitivity analyses were run using only the plausible reporters (*n* = 61), which produced findings consistent with the full sample. Dietary intakes of median overall energy and macronutrient intakes were higher among plausible reporters than in the full sample, which would be expected following the removal of under-reporters. Despite this the overall dietary pattern of inadequate energy, carbohydrate intake and insufficient micronutrient consumption was consistent (Supplementary Table S1); for example, median carbohydrate intake amongst plausible reporters was 3.8 g/kg/d [IQR 3.4, 4.3] compared with 3.2 g/kg/d [IQR 2.6, 3.8] reported within the full sample, remaining below the 4–8 g/kg/d recommendation. These analyses did not materially alter the study’s conclusions, therefore dietary intake data was reported for all 139 participants.

### 3.2. Overall Team Sport Athletes Nutrition Knowledge

The full cohort (*n* = 354) had a mean total nutrition knowledge score of 48.64 ± 12.82%, falling within the “poor” classification (Table 2). Most participants (53.1%) had “poor” nutrition knowledge; only 9.1% achieved “good” or “excellent” scores. Nutrition knowledge category was not associated with sex, playing level, or sport. Total nutrition knowledge score was not associated with age or years playing, although a weak positive association was observed with weekly training hours (*r* = 0.118, *p* = 0.027).

### 3.3. Comparison of Nutrition Knowledge Between Groups

**Figure 1:**
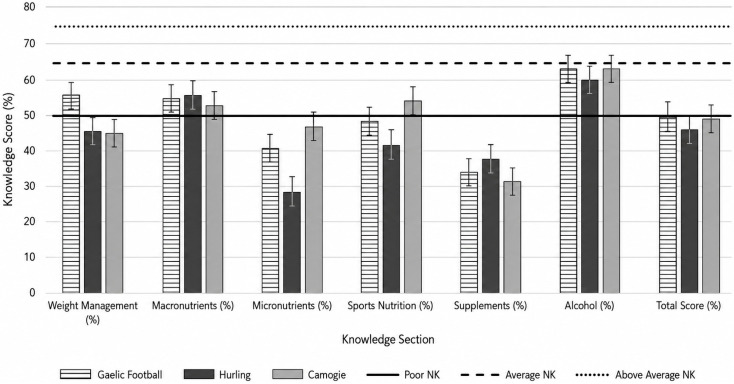
Nutritional knowledge of Gaelic players stratified by sport (Gaelic football (*n* = 122), hurling (*n* = 6) and camogie (*n* = 11)) within the dietary reporters sample. NK, nutritional knowledge. Scores are given as mean ± pooled within-group SD (%). Horizontal lines represent knowledge scoring criteria; 0–49 (poor), 50–64 (average), 65–100 (above average). No significant difference (*p* < 0.05) was observed between sports, as determined by one-way ANOVA.

Nutrition knowledge scores did not differ meaningfully between the full cohort and the dietary subsample (Table 3). Across all groups, scores were weakest within the supplementation, micronutrient, and sports nutrition subsections. No significant differences in nutrition knowledge were observed between Gaelic football, hurling, and camogie (Figure 1).

Nutrition knowledge did not differ by sex or playing level overall; however, females scored higher than males within the micronutrient subsection (47.9 ± 13.2% vs. 38.2 ± 19.9%; *p* = 0.002; Cohen’s d = 0.53), and elite players scored higher than sub-elite players in the macronutrient subsection (59.5 ± 14.1% vs. 53.5 ± 17.5%; *p* = 0.046; Cohen’s d = 0.36) (Table 4) [23].

### 3.4. Demographic Questionnaire

Of the 139 dietary intake reporters, only 6.5% had ever completed formal studies in nutrition. The majority of participants (67.6%) thought sporting organisations should provide members access to nutritional info and nutritionists/dieticians, while 23% thought sporting organisations should provide nutritional information only, and 5.8% answered “neither of above” (3.6% gave no answer). When asked “Does club provide you with access to nutrition info or dietician/nutritionist”, 64.7% of participants had no access to either nutritional information or a nutritionist/dietician, 21.6% had access to nutritional information only, and 12.9% had access to nutritional information and a nutritionist/dietician (0.7% gave no answer). A significant difference was observed within the sports nutrition subsection mean scores of the NSKQ between participants who had access to both nutritional information and nutritionists/dieticians (58.3 ± 12.5%) and participants who had access to neither (45.0 ± 18.9%) (*p* = 0.008). No other significant difference between the participants’ total scores or subsection scores and level of access to nutritional information and nutritionists/dieticians was found.

Participants were also asked to rank what nutritional resource would be most useful to them; the top ranked resource was “access to nutritional info relevant to sports/training nutrition” (46.8%), though a close second was “individual consultations by nutritionists/dieticians” (40.3%). The lowest ranked nutritional resource by participants was joint “access to group presentations by nutritionists/dieticians” and “cooking classes” (5.8%).

### 3.5. Dietary Intake

Dietary intake was assessed against population and sports nutrition recommendations. Overall, 84.9% of participants under-consumed energy (median deficit 755 kcal/d [IQR −1085, −115]), predominantly due to insufficient carbohydrate intake; only 20.9% met the 4–8 g/kg/d recommendation. Protein and fat intakes were generally adequate; water and most micronutrients fell within recommended ranges. However, few participants met recommendations for vitamin D (5.8%) or fibre (12.2%), and no female participant met the iron recommendation of 14.8 mg/d (Table 5 and Table 6).

When stratified by nutrition knowledge category, participants with “average or above” knowledge were more likely to meet energy and fibre recommendations than those with poor knowledge; no other significant differences were observed, although a trend toward higher carbohydrate adequacy was noted in the higher knowledge group (26.8% vs. 14.7%, *p* = 0.08). However, in logistic regression models adjusted for sex, playing level, and training hours, the association with meeting energy recommendations was no longer significant (OR = 0.984 per 1% increase; 95% CI 0.948–1.022; *p* = 0.406), and for fibre, higher knowledge was unexpectedly associated with a lower likelihood of meeting recommendations (OR = 0.922; 95% CI 0.878–0.968; *p* = 0.001) (Supplementary Table S2).

## 4. Discussion

The aim of this study was to assess the nutrition knowledge and dietary intake of Gaelic team sport athletes while exploring any association between these variables. The key findings indicated that nutrition knowledge and dietary intake of players was sub-optimal. NSKQ total scores fell within the “poor” classification, while sub-optimal dietary intake was characterised by insufficient intakes of overall energy, carbohydrate, vitamin D and fibre. While previous research indicated higher nutrition knowledge was modestly associated with a greater likelihood of team sport athletes meeting certain dietary intake recommendations such as increased intakes of fruits, vegetables, and protein [5], few participants demonstrated “good” or “excellent” nutrition knowledge and/or met the majority of dietary intake recommendations for the demands of their sport.

The findings of this study align closely with previous research in team sport athletes [5]. Participants demonstrated poor overall nutrition knowledge, with specific nutrition knowledge deficits observed within the sports nutrition, micronutrients and supplementation subsections. The observed pattern of sub-optimal dietary intake characterised by inadequate energy and carbohydrate intake alongside adequate protein and fat consumption reflects the trends observed across multiple team sport athletes populations [5]. Furthermore, the modest association between nutrition knowledge and dietary intake, the negligible differences between elite and sub-elite players, and the low prevalence of adequate vitamin D intake observed in our study support previous findings in team sport athletes [5].

### 4.1. Nutritional Knowledge

Overall, participants were observed to have “poor” nutrition knowledge. Nutrition knowledge scoring did not differ meaningfully between groups when stratified by sex, sport, or playing level. While some significant differences between groups were observed, deficits in nutrition knowledge were evident across all groups, which suggests that observed deficits in nutrition knowledge may be systemic rather than group-specific. Female athletes scored significantly higher within the micronutrient subsection compared to male athletes; this may reflect greater exposure to targeted female health messaging/marketing surrounding iron, calcium and bone health, which appears to have had a demonstrable impact on this specific knowledge domain. However, it is important to note that scoring within this subsection was categorised as “poor” for both males and females, indicating that despite this relative advantage, overall micronutrient knowledge is still inadequate across sex.

Elite athletes scored significantly higher within the macronutrient subsection compared to sub-elite athletes. One possible explanation is greater exposure to nutritional support, or environmental influences from coaches and teammates, or even the food that is provided for them. Moreover, elite players may have greater health-seeking behaviours due to the level of competition they participate in. Although elite players demonstrated meaningfully higher macronutrient knowledge than their sub-elite counterparts, both groups’ mean scores remained within the “average” classification, suggesting that even players with greater resources and support may not achieve “good” or “excellent” nutrition knowledge without targeted intervention. A small positive association between nutrition knowledge and training hours was observed; however, the strength of this relationship was weak and unlikely to be practically meaningful. It could however reflect that team sport athletes who train more, may be more engaged athletes, potentially increasing their general exposure to sports nutrition related information from various sources including teammates and coaches.

These findings align with the existing literature of Gaelic team sport athletes populations, with a study by Murphy and O’Reilly (2021) [6] observing a mean nutrition knowledge score of 48.8% within elite and sub-elite hurlers using the validated Sports Nutrition Knowledge Questionnaire. While the nutrition knowledge questionnaires differed between the current study and the work of Murphy and colleagues, both studies observed no significant difference in total nutrition knowledge between elite and sub-elite players, reinforcing that poor nutrition knowledge is systemic across playing levels. Similarly, both studies highlighted consistent gaps in specific nutrition knowledge around supplements and sports nutrition. Similarly, access to qualified nutrition support was associated with higher nutrition knowledge scores in both cohorts, though neither study found this translated meaningfully into overall nutrition knowledge scoring [6].

The findings reported by McCrink et al. (2021) [8] further support and reinforce the results of this study. Both studies utilised the validated NSKQ to assess nutrition knowledge within a unique and under-researched cohort. Whilst our study has a larger and more diverse sample encompassing male and female athletes across Gaelic football, hurling, and camogie, similar nutrition knowledge was observed. Mean nutrition knowledge score observed in both studies fell within the “poor” classification, with the present study reporting higher total nutrition knowledge scores (48.64 ± 12.82% vs. 40.2 ± 12.4%). Differences in sample size, participant characteristics and study population may partly explain this difference when compared to the significantly smaller sample (*n* = 24) of male Gaelic footballers in the McCrink study. Despite the variation in sample size, diversity and overall nutrition knowledge score, both studies identified micronutrients, sports nutrition and supplements as the weakest NSKQ subsections, again reinforcing that these are consistent nutrition knowledge gaps among Gaelic team sport athletes [8].

Deficits in nutrition knowledge within these specific areas may have real-world implications for athletes; for example, a lack of nutrition knowledge around supplements could lead to team sport athletes potentially consuming supplements which may be ineffective, harmful or even banned within their sport. Additionally, within this study and the McCrink paper, the majority of participants expressed a preference for individual consultations with qualified nutritionists over group presentations, highlighting a shared desire for tailored personalised nutrition support [8].

The majority of participants reported having no access to nutrition information or a qualified nutritionist/dietitian through their club, despite most participants believing that sporting organisations should provide access to these resources. Participants identified access to sport-specific nutrition information and individual consultations with a nutritionist/dietitian as the most valuable forms of nutrition support. Participants who reported access to both nutrition information and a nutritionist/dietitian demonstrated significantly higher scores within the sports nutrition subsection than those with access to neither. Similar findings have been reported in previous research suggesting that structured nutrition support delivered by appropriately qualified practitioners can improve specific nutrition knowledge [15]. However, this difference was limited to the sports nutrition subsection, with no significant differences observed in overall nutrition knowledge or the remaining subsections. These findings suggest that access to nutrition support, as currently provided within the participating sporting organisations, may contribute to improvements in specific areas of sports nutrition knowledge but may not be associated with broader nutritional knowledge. Previous research suggests that improving nutritional knowledge is likely to require a multifaceted approach. Resource-related barriers such as time and cost, along with individual constraints such as motivational, or environmental factors may influence the effectiveness of nutrition education interventions [24]. Future research should investigate how the availability, quality, frequency, and mode of delivery of nutrition support influence athletes’ nutrition knowledge and dietary behaviours.

### 4.2. Dietary Intake

The findings of this study are broadly consistent with previous research in Gaelic team sport athletes [8,10]. Participants reported energy intakes substantially below estimated requirements (median deficit 755 kcal/d), and carbohydrate consumption was below current sports nutrition recommendations. However, these findings should be interpreted with caution; over half of the dietary sample was classified as under-reporters at the individual level (56.1%). Although sensitivity analyses confined to plausible reporters (*n* = 61) produced findings consistent with the full sample for all key dietary variables (Supplementary Table S1), the possibility remains that some of the observed energy and carbohydrate deficits reflect systematic under-reporting rather than true dietary inadequacy alone. Reported energy intake was below estimated requirements and appeared to stem predominantly from inadequate carbohydrate intake (3.2 g/kg/d), with only 21% of participants meeting the current sports nutrition recommendations for carbohydrate intake (4–8 g/kg/d) [11]. Given the intermittent high-intensity nature of GAA sports, insufficient acute carbohydrate intake may significantly reduce sports performance, by potentially decreasing power output, work capacity, repeated sprint ability, time to fatigue and even increasing the risk of hypoglycaemia [12,25]. Chronic low carbohydrate availability may also contribute to low energy availability (LEA) [13], leading to potential downstream negative health effects on outcomes such as endocrine function, bone health and immune status [26].

In contrast, median protein (1.6 g/kg/d) and fat intake (32.0% EI) were generally within recommended dietary intake ranges, although it is important to note that only 51.1% of participants met protein recommendations with 17 participants (23.94% of those who met protein recommendations) actually exceeding the upper threshold of 2.2 g/kg/d. A pattern of adequate protein intake but insufficient carbohydrate consumption has previously been observed in team sport athletes populations [8] and may reflect prevailing societal beliefs and food product marketing promoting protein for performance and recovery. These patterns may also be partially reflected by participants scoring “poor” within the sports nutrition subsection which focuses on fuelling for sport vs. “average” in the macronutrient section where questions are more related to the macronutrient content of foods.

Micronutrient intakes were notably low for vitamin D, fibre, and iron. Only 5.8% and 12.2% of participants met recommendations for vitamin D and fibre, respectively, and no female participant met the iron recommendation. These apparent inadequacies should be interpreted with caution, as they may partly reflect under-reporting. Nevertheless, sub-optimal vitamin D intakes are not unexpected given the limited food sources naturally rich in vitamin D and the low ultraviolet B exposure typical of northern latitudes [21]; inadequate dietary intake may compound the risk of deficiency in this population [27]. Indeed, we have previously observed a high prevalence of vitamin D insufficiency in male and female Gaelic footballers [27]. Of particular concern, vitamin D deficiency in athletes has been associated with impaired muscle function, increased injury risk, increased risk of stress fractures, and compromised immune health [12]. Observed low fibre intakes are likely reflective of insufficient consumption of whole grains, fruits, and vegetables. Whilst fibre is not directly ergogenic, its role in gastrointestinal health, glycaemic regulation, and cardiometabolic health is well established [19]. Practically, increasing fibre intake may concurrently improve team sport athletes overall dietary intake quality and micronutrient density [28]. The observation that no female participant met the iron recommendation is notable, although it should be interpreted with caution given the small number of females with dietary data (*n* = 36) and the high prevalence of under-reporting in this group. Iron deficiency is common in female athlete populations and has been associated with impaired exercise capacity and immune function [12]. Low energy and carbohydrate intakes may contribute to this apparent inadequacy, as iron-rich foods such as red meat and fortified cereals may be consumed in insufficient quantities.

### 4.3. Relationship Between Nutrition Knowledge and Dietary Intake

Given the cross-sectional nature of this study, the observed associations between nutrition knowledge and dietary intake should be interpreted with caution, as causal relationships cannot be established. Nevertheless, participants with “average and above” nutrition knowledge were observed to be more likely to meet energy and fibre recommendations; however, no consistent differences were observed across most dietary intake variables. It is also important to highlight that since so few players in this study achieved a score reflective of ‘good’ or ‘excellent’ nutrition knowledge, this may have impacted our findings by limiting the ability to detect associations between higher nutrition knowledge and DI, as the comparison was effectively between “poor” and “average” knowledge. The observed associations were modest, limited to only two of the dietary outcomes examined, and were substantially attenuated in the adjusted logistic regression models (Supplementary Table S2). These results suggest that nutrition knowledge alone may not strongly determine dietary behaviour change [29]. It should be noted that the cross-sectional design does not permit conclusions about whether greater nutrition knowledge leads to better dietary intake, or whether both are simply markers of other unmeasured factors such as access to nutrition support, greater engagement with sport, or educational background. The following discussion of potential mechanisms is therefore speculative and was not directly tested in the present study. The influences of dietary behaviour are complex and multifaceted and additionally all GAA sports are amateur, adding an additional layer of complexity; as these athletes often must balance training/competition with work or study. Previous research in team sport athletes has demonstrated weak-to-moderate positive associations between nutrition knowledge and fruit and vegetable intake [30,31], as well as between nutrition knowledge and energy and carbohydrate intake [32]. While nutritional counselling and education interventions was associated with improvements in both nutrition knowledge and dietary intake among athletes, dietary changes have often fallen short of optimal levels [33,34], underscoring the need for behaviour change frameworks to support translation of knowledge into practice. Other practical barriers which may limit the translation of nutrition knowledge into nutritional practice for this population include environmental constraints, food availability, time, habits, cultural norms, and motivation [24]. Regardless, improving nutrition knowledge remains a worthwhile objective, as nutrition knowledge remains a key component of enhancing dietary behaviour. While these results highlight that nutrition knowledge alone is not sufficient to ensure dietary adequacy, these findings support the growing consensus that nutritional interventions should incorporate behaviour change frameworks alongside education [35,36]. The preference expressed by participants for tailored nutrition information and individual consultation further supports the need for an effective educational intervention to meet these demands.

The adjusted logistic regression models indicated that the bivariate association between nutrition knowledge and meeting energy recommendations was no longer significant after accounting for covariates; sex, playing level, and training hours. An unexpected inverse association emerged for fibre, with higher nutritional knowledge associated with lower rates of meeting fibre recommendations. This finding should be interpreted cautiously due to the small sample of participants who actually managed to meet fibre recommendations (*n* = 17), leaving this model susceptible to instability. Nonetheless, these findings highlight the multifactorial nature of dietary behaviour, reinforcing that nutrition knowledge alone may only be a modest determinant of dietary adequacy.

### 4.4. Practical Applications

The practical implications discussed below should be considered in light of the study’s methodological limitations. The dietary intake data were derived from a subsample with a high prevalence of under-reporting, and the cross-sectional design precludes causal inferences about the effects of knowledge on behaviour. The following suggestions represent reasonable directions based on the present findings, but definitive intervention strategies should be informed by future longitudinal and intervention-based research. The main findings observed in this study were the sub-optimal nutrition knowledge and dietary intake of the participants. While both total and subsection nutrition knowledge scores were sub-optimal, from a practical standpoint the nutrition knowledge findings are useful to identify which areas future nutritional interventions could target. Sports nutrition, supplementation and micronutrients highlight priority target areas for educational interventions, with particular emphasis on translating macronutrient knowledge into practical fuelling strategies. Addressing supplement misconceptions is essential as poor nutrition knowledge in this area may expose athletes to ineffective, harmful, or inadvertent consumption of banned substances. An accessible entry point may be to build upon areas of relative strength such as alcohol and weight management before introducing more complex topics. The observed preference for individual nutrition consultations and tailored nutrition information over group presentations, suggests that one-to-one, including scalable digital platforms, may enhance engagement.

The key dietary intake finding observed was the inadequate energy intake of this cohort, stemming primarily from insufficient carbohydrate consumption. Addressing this energy deficit by increasing carbohydrate intake to meet the recommended range of 4–8 g/kg/d could support real-world improvements in performance and could be achieved through the addition of carbohydrate rich meals or snacks aligned with sporting demands. Low intakes of vitamin D and fibre were observed, with only 5.8% and 12.2% of participants meeting recommendations, respectively. The low prevalence of adequate vitamin D intake suggests that supplementation strategies, particularly during winter months or periods of limited sunlight exposure, are warranted in line with current government guidelines for the general population (10 µg/d in UK, [21]). The observation that no female participant met the recommendation for iron highlights a need for targeted strategies to improve iron intake among female GAA team sport athletes, including consumption of iron-rich foods such as lean red meat, fortified cereals, and legumes, alongside vitamin C to enhance absorption. Increasing fibre intake to 30 g/day [19] through whole-food sources such as an additional meal consisting of a whole grain carbohydrate source (whole grain bread, rice, pasta) may simultaneously address multiple dietary inadequacies.

### 4.5. Limitations and Strengths

The main methodological limitation of this study is the cross-sectional observational design. This is a common limitation in existing studies examining nutrition knowledge and dietary intake in team sport athletes, which prevents causal interpretation. A longitudinal intervention with pre- and post-measures is a more appropriate design to identify if changes in nutrition knowledge translate into altered dietary intake behaviour; additionally, accounting for external influences by incorporating a control group would further enhance causal inference.

In the assessment of nutrition knowledge, one limitation of the NSKQ is that it relies solely on written descriptions, which may be unclear to some athletes, whereby adding visual depictions of portion sizes could improve clarity and accessibility.

Additionally, the use of a 4-day food diary to assess dietary intake also presents limitations, including participant burden and compliance, which may have contributed to the lower completion rate for dietary assessment. All participants were provided with the same blank 4-day food diary template and standardised instructions, including an example of a completed food diary. To maximise the completion rate, food diaries were accepted in multiple formats; while this may open up the possibility for potential differences in reporting, no notable differences were apparent across formats. This could further have introduced selection bias, potentially limiting generalisability. A lower proportion of female participants completed the 4-day food diary compared to males (25.9% vs. 47.9%), which may further limit the generalisability of the dietary intake findings to female GAA team sport athletes. Apart from the lower completion rate for dietary assessment by female participants, dietary reporters and non-reporters did not differ significantly on any other measured characteristic (age, playing level, training hours, years playing, education, and access to nutrition support; all *p* > 0.05), suggesting limited selection bias on these variables. The high prevalence of under-reporting at the individual level (56.1%) is a further limitation; although, sensitivity analyses confined to plausible reporters did not materially alter the study’s conclusions. Furthermore, dietary intake was compared to general population and sports nutrition recommendations rather than to Gaelic-specific guidelines, which are currently unavailable. The classifications of inadequacy reported here should therefore be interpreted as indicative rather than definitive.

Another limitation is that energy availability could not be directly assessed as exercise energy expenditure was not measured. Therefore, although reported energy intake was compared with estimated energy requirements and discussed in the context of potential low energy availability, definitive conclusions cannot be drawn. Future research could incorporate direct or validated estimates of exercise energy expenditure to permit a more comprehensive assessment of energy availability in Gaelic team sport athletes. Other limitations included the awareness of being monitored which may alter dietary behaviour, recall-bias and estimation errors, although sensitivity analyses suggested minimal impact on the overall findings.

Despite these limitations, the use of the validated NSKQ is a major strength. Each subsection can be administered and interpreted independently, which presents a practical advantage for evaluating specific areas of nutrition knowledge. Notwithstanding the aforementioned limitations of 4-day food diaries, utilisation of estimated dietary records over a 3–4-day period have been previously identified within the literature as a commonly used method of dietary assessment amongst athletes. This approach is considered accurate for assessment at individual and group level and is more convenient for time-constrained athletes than other methods [37]. Additionally, when assessing dietary intake, misreporters were equated for by the application of the revised Goldberg cut-off method. A major strength of this study was the adequately powered sample size and representation of traditional Irish Gaelic team sports across different playing levels and sex, providing a valuable insight into the nutrition knowledge and dietary intake of Gaelic players.

## 5. Conclusions

Our findings indicate that Gaelic team sport athletes have generally poor nutrition knowledge and possible dietary inadequacies, characterised by insufficient consumption of energy and carbohydrate in relation to the demands of their sport, alongside inadequate vitamin D and fibre intake. However, the translation of nutrition knowledge into dietary behaviour appears limited and is likely influenced by multiple factors not captured in the present study. While athletes with higher nutrition knowledge were more likely to meet energy and fibre recommendations in unadjusted analyses, these associations were modest, limited to a subset of outcomes, and substantially attenuated after adjustment for confounders. The gap between knowing and doing remains substantial. These findings highlight the need for interventions that combine nutrition education with behaviour change strategies. Future research using longitudinal and intervention-based designs with more robust dietary assessment methods is needed to confirm these observations and to examine how improvements in nutrition knowledge may translate into sustained dietary change.

## 6. Novelty Statement

This study contributes to the existing literature by providing initial evidence on the relationship between nutritional knowledge and dietary intake in a cohort of traditional Irish Gaelic team sport athletes. Further research is warranted to explore these relationships using rigorous methodologies and to evaluate strategies aimed at enhancing nutritional knowledge and subsequent dietary behaviours.

## Figures and Tables

**Table 1:** Demographic characteristics.

Characteristic	Total Cohort (*n* = 354)	Males (*n* = 215)	Females (*n* = 139)
	Mean ± SD	Mean ± SD	Mean ± SD
Age (years)	25.34 ± 5.27	26.00 ± 5.70	24.31 ± 4.81
Weight (kg)	75.67 ± 12.16	82.45 ± 8.47	65.05 ± 9.07
Height (m)	1.75 ± 0.10	1.82 ± 0.06	1.66 ± 0.07
BMI (kg/m^2^)	24.45 ± 2.57	24.97 ± 2.28	23.63 ± 2.79
Weekly Training (h)	7.55 ± 3.11	8.09 ± 3.28	6.72 ± 2.64
BMR (kcal/day)	1800 ± 232	1929 ± 127	1635 ± 148
EI:BMR Ratio	1.29 ± 0.41	1.31 ± 0.35	1.09 ± 0.23
Playing Level:	*n* (%)	*n* (%)	*n* (%)
Elite (Intercounty)	133 (37.6)	86 (40.0)	47 (33.8)
Sub-elite (Club/University)	221 (62.4)	129 (60.0)	92 (66.2)
Sport:	*n* (%)	*n* (%)	*n* (%)
Gaelic Football	271 (76.6)	187 (87.0)	84 (60.4)
Hurling	28 (7.9)	28 (13.0)	-
Camogie	55 (15.5)	-	55 (39.6)
Dietary Intake:	*n*	*n*	*n*
Reported Dietary Intake	139	103	36
Plausible reporters	61	50	11
Misreporters	78	53	25
Under-Reporters	74	49	25
Over-Reporters	4	4	-

kg, kilograms; BMR, basal metabolic rate; EI:BMR, energy intake-to-BMR ratio; kcal/day, energy per day.

**Table 2:** Nutritional knowledge categorisation of players stratified by sex, playing level and sport.

NSKQ Nutritional Knowledge Category	Total Cohort (*n* = 354) *n* (%)	
Poor Knowledge (0–49%)	188 (53.1%)	
Average Knowledge (50–64%)	134 (37.8%)	
Good Knowledge (65–75%)	25 (7.1%)	
Excellent Knowledge (76–100%)	7 (2.0%)	
Stratified Poor Knowledge (0–49%) or Average and Above Knowledge (50–100%)	Poor *n* (%)	Average and Above *n* (%)
Total Cohort (*n* = 354)	188 (53.1%)	166 (46.9%)
NSKQ and Food Diaries (*n* = 139)	68 (48.9%)	71 (51.1%)
Sex:	*n* (%)	*n* (%)
Male (*n* = 215)	119 (55.3%)	96 (44.7%)
Female (*n* = 139)	69 (49.6%)	70 (50.4%)
Playing Level:	*n* (%)	*n* (%)
Elite (Intercounty) (*n* = 133)	64 (48.1%)	69 (51.9%)
Sub-elite (Club/University) (*n* = 221)	124 (56.1%)	97 (43.9%)
Sport:	*n* (%)	*n* (%)
Gaelic Football (*n* = 271)	144 (53.1%)	127 (46.9%)
Hurling (*n* = 28)	19 (67.9%)	9 (32.1%)
Camogie (*n* = 55)	25 (45.5%)	30 (54.5%)

Chi-squared tests indicated no evidence of an association between stratified nutritional knowledge category and sex (*p* = 0.293), playing level (*p* = 0.145), or sport (*p* = 0.154) within the total cohort.

**Table 3:** Nutritional knowledge of GAA team sport athletes.

NSKQ Total and Subsections *(n* = No. Questions)	Original Cohort (*n* = 354) % Mean ± SD	Non-Dietary Intake Reporters (*n* = 215) % Mean ± SD	Dietary Intake Reporters (*n* = 139) % Mean ± SD	NSKQ Scoring Classification	*p*-Value
Weight management (*n* = 12)	54.9 ± 16.7	54.9 ± 15.5	54.9 ± 18.7	“Average” (50–64%)	0.973
Macronutrients (*n* = 30)	54.1 ± 16.8	53.2 ± 16.9	55.5 ± 16.6	“Average” (50–64%)	0.213
Micronutrients (*n* = 13)	40.3 ± 19.6	39.9 ± 20.2	40.7 ± 18.9	“Poor” (0–49%)	0.708
Sports Nutrition (*n* = 12)	46.2 ± 17.5	44.7 ± 17.3	48.4 ± 17.7	“Poor” (0–49%)	0.049 *
Supplementation (*n* = 12)	32.5 ± 17.9	31.6 ± 17.6	33.8 ± 18.2	“Poor” (0–49%)	0.276
Alcohol (*n* = 8)	60.4 ± 17.6	58.4 ± 17.7	63.3 ± 17.1	“Average” (50–64%)	0.011 *
Total (*n* = 87)	48.6 ± 12.8	47.8 ± 12.6	49.9 ± 13.1	“Poor” (0–49%)	0.123

* *p*-value determined by independent *t*-tests comparing dietary intake non-reporters (*n* = 215) and dietary intake reporters (*n* = 139).

**Table 4:** Dietary intake reporters’ nutritional knowledge.

NSKQ	Dietary Intake Reporters (*n* = 139)						
	Mean ± SD	Elite (*n* = 46)	Sub-Elite (*n* = 93)	*p*-Value *	Male (*n* = 103)	Female (*n* = 36)	*p*-Value *
Weight Management (%)	54.9 ± 18.7	56.2 ± 18.1	54.2 ± 19.1	0.565	55.1 ± 18.6	54.2 ± 19.4	0.799
Macronutrients (%)	55.5 ± 16.6	59.5 ± 14.1	53.5 ± 17.5	0.046	56.4 ± 17.8	53.0 ± 12.5	0.213
Micronutrients (%)	40.7 ± 18.9	42.0 ± 16.1	40.1 ± 20.1	0.587	38.2 ± 19.9	47.9 ± 13.2	0.002
Sports Nutrition (%)	48.4 ± 17.7	50.7 ± 13.5	47.3 ± 19.4	0.229	48.0 ± 17.8	49.8 ± 17.5	0.602
Supplements (%)	33.8 ± 18.2	32.4 ± 13.7	34.4 ± 20.1	0.497	33.7 ± 17.0	33.8 ± 21.5	0.988
Alcohol (%)	63.3 ± 17.1	62.8 ± 18.5	63.6 ± 16.4	0.795	62.5 ± 17.1	65.6 ± 17.0	0.347
Total Score (%)	49.9 ± 13.1	51.8 ± 10.6	49.0 ± 14.1	0.246	49.8 ± 13.7	50.4 ± 11.2	0.791
Total Mean Score Knowledge Category	Poor	Average	Poor	-	Poor	Average	-

%, percentage. Values are given as mean scores ± SD. * *p*-value determined by independent *t*-tests.

**Table 5:** Mean Energy, macronutrient, water and alcohol intake of the dietary intake sample, and percentage of sample and the different knowledge categories meeting DRVs.

Average Daily Intake		Dietary Intake Sample Intake Median (IQR)	DRV	% Meeting DRVs			
				Total Sample (*n* = 139) (%)	Poor Knowledge (*n* = 68) (%)	Average and Above Knowledge (*n* = 71) (%)	*p*-Value ^b^
Total Energy	Kcal/d	2161.0 (1330.8, 2801.3)	$x\bar{=}2916.19$ ^a^	21 (15.1%)	6 (8.8%)	15 (21.1%)	0.043 *
Energy Deficit	Kcal/d	−755.2 (−1085.4, −114.9)	-	-	-	-	-
Carbohydrate	g/kg/d	3.2 (2.6, 3.8)	4–8 ^b a^	29 (20.9%)	10 (14.7%)	19 (26.8%)	0.080
Protein	g/kg/d	1.6 (1.2, 2.1)	1.6–2.2 ^b^	71 (51.1%)	30 (44.1%)	41 (57.7%)	0.108
Fat	% EI	32.0 (29.0, 36.0)	20–35 ^c^	99 (71.2%)	48 (70.6%)	51 (71.8%)	0.871
Saturated Fat	% EI	12.0 (10.0, 14.0)	≤10	38 (27.3%)	19 (27.9%)	19 (26.8%)	0.876
Fibre	g/day	20.5 (15.3, 26.8)	30 ^d^	17 (12.2%)	2 (2.9%)	15 (21.1%)	0.001 *
Water	L/day	2.3 (1.3, 3.3)	2–5 ^b^	79 (56.8%)	38 (55.9%)	41 (57.7%)	0.824
Alcohol	g/day	0.0 (0.0, 10.7)	0 g ^b^	88 (63.3%)	42 (61.8%)	46 (64.8)	0.712

DRV, dietary reference value. Average and above nutrition knowledge includes average, good and excellent knowledge. For protein, the % meeting DRVs includes participants exceeding the upper bound of 2.2 g/kg/d. %EI percentage of energy intake. ^a^ BMR × PAL (1.6). ^b^ [11]. ^c^ [12]. ^d^ [19]. * Significant difference between groups (*p* < 0.05), **^b^** *p*-value determined by Chi-squared test.

**Table 6:** Mean micronutrient intake of dietary intake reporters, and percentage of total sample and the different knowledge categories meeting DRVs.

Average Daily Intake		DI Sample Intake Median (IQR)	RNI	% Meeting DRVs			
				Total Sample (*n* = 139) (%)	Poor Knowledge (*n* = 68) (%)	Average and Above Knowledge (*n* = 71) (%)	*p*-Value ^b^
**Vitamin A**	μg/day	*M*: 655 (417, 981) *F*: 575 (357, 804)	*M*: 700 ^a^/*F*: 600 ^a^	65 (46.8%)	30 (44.1%)	35 (49.3%)	0.541
**Vitamin C**	mg/day	69.0 (39.3, 112.0)	40 ^a^	102 (73.4%)	44 (64.7%)	58 (81.7%)	0.024 *
**Vitamin D**	μg/day	3.2 (1.9, 6.0)	10 ^d^	8 (5.8%)	3 (4.4%)	5 (7%)	0.719
**Vitamin E**	mg/day	*M*: 6.9 (5.4, 10.1) *F*: 5.7 (4.3, 7.5)	*M*: >4 ^a^/*F*: >3 ^a^	123 (88.4%)	58 (85.3%)	65 (91.5%)	0.248
**Iron**	mg/day	*M*: 12.2 (8.2, 15.8) *F*: 8.7 (6.4, 11.0)	*M*: 8.7 ^a^/*F*: 14.8 ^a^	75 (54.0%)	35 (51.5%)	40 (56.3%)	0.565
**Calcium**	mg/day	770.0 (591.0, 1046.0)	700 ^a^	79 (56.8%)	33 (48.5%)	46 (64.8%)	0.053
***n*** **-3 PUFA**	g/day	0.76 (0.46, 1.20)	0.45 ^c^	106 (76.3%)	53 (77.9%)	53 (74.6%)	0.648

RNI, reference nutrient intake. Average and above nutrition knowledge includes average, good and excellent knowledge. %EI, percentage of energy intake, *n*-3 PUFA, omega-3 polyunsaturated fatty acids. M = male, F = female. ^a^ [20]. ^c^ [18]. ^d^ [21]. * Significant difference between groups (*p* < 0.05), **^b^** *p*-value determined by Chi-squared test or Fisher’s Exact Probability test.

## Data Availability

The data presented in this study are available on request from the corresponding author.

## Supplementary Materials

The following supporting information can be downloaded at: https://www.mdpi.com/article/10.3390/nu18142259/s1, Table S1: Sensitivity analysis comparing dietary intake between the full dietary sample (*n* = 139) and plausible reporters (*n* = 61). Values are median [IQR]; Table S2: Logistic regression models predicting meeting of energy and fibre recommendations.

## Abbreviations

The following abbreviations are used in this manuscript:

ANOVA	Analysis of Variance
BMR	Basal Metabolic Rate
BMI	Body Mass Index
CI	Confidence Interval
DRV	Daily Recommended Value
EI	Energy Intake
GAA	Gaelic Athletic Association
IQR	Interquartile Range
LEA	Low Energy Availability
NSKQ	Nutrition for Sport Knowledge Questionnaire
PAL	Physical Activity Level
PUFA	Polyunsaturated Fatty Acids
*n*-3 PUFA	Omega-3 Polyunsaturated Fatty Acids
REDCap	Research Electronic Data Capture
RNI	Reference Nutrient Intake
SD	Standard Deviation
SPSS	Statistical Package for the Social Sciences
UK	United Kingdom
%EI	Percentage of Energy Intake
