# Supplementary material for: Sub-Optimal Nutritional Knowledge and Dietary Intake in Gaelic Team Sport Athletes: Limited Associations Between Knowledge and Dietary Adequacy
Source: Nutrients. 2026 Jul 10;18(14):2259. doi: 10.3390/nu18142259 (PMC13414835; doi:10.3390/nu18142259)
Supplement: Supplementary file 1 [file nutrients-18-02259-s001.zip › nutrients-4408778-supplementary.pdf]

Supplementary Tables

Table S1. Sensitivity analysis comparing dietary intake between the full dietary sample (*n*=139) and plausible reporters (*n*=61). Values are median [IQR].

Variable	Recommendations	Full Sample (*n*=139) Median [IQR]	Plausible Reporters (*n*=61) Median [IQR]
**Energy (kcal/d)**	2916[a]	2161 [1331, 2801]	2801 [2606, 3084]
**CHO (g/kg/d)**	4-8[b]	3.2 [2.6, 3.8]	3.8 [3.4, 4.3]
**Protein (g/kg/d)**	1.6-2.2[b]	1.6 [1.2, 2.1]	2.1 [1.8, 2.4]
**Fat (% EI)**	20-35[c]	32.0 [29.0, 36.0]	32.0 [29.0, 36.5]
**Fibre (g/d)**	30[d]	20.5 [15.3, 26.8]	26.8 [22.1, 29.8]
**Vitamin D (µg/d)**	10[e]	3.2 [1.9, 6.0]	4.8 [2.9, 8.4]
**Iron (mg/d) (Female)**	14.8[f]	8.7 [6.4, 11.0]	8.5 [6.5, 10.7]

[a] BMR × PAL (1.6). [b] Collins et al., 2021. [c] Thomas et al., 2016. [d] SACN, 2015. [e] SACN, 2016. [f] COMA, 1991.

Table S2. Logistic regression models predicting meeting of energy and fibre recommendations.

Predictor	Energy OR [95% CI]	P Value	Fibre OR [95% CI]	P Value
**NK Total Score (%)**	0.984 [0.948, 1.022]	0.406	0.922 [0.878, 0.968]	0.001
**Sex (Female vs. Male)**	7.110 [0.896, 56.422]	0.063	6.811 [0.819, 56.679]	0.076
**Level (Elite vs. Sub-elite)**	1.064 [0.383, 2.960]	0.905	0.522 [0.148, 1.843]	0.313
**Training Hours (weekly)**	0.904 [0.779, 1.048]	0.180	1.009 [0.841, 1.211]	0.925

Note: Both models were adjusted for sex, playing level, and weekly training hours. Sport and access to nutrition support were excluded due to quasi-complete separation in the fibre model (0 or near 0 cell counts in some categories). Odds ratios are per 1% increase in nutrition knowledge total score and per additional hour of weekly training. Full model coefficients (B, SE, Wald) are available on request from the corresponding author.
